# Women Offenders Under Community Supervision: Comparing the Profiles of Returners and Non-Returners to Federal Prison

**DOI:** 10.3389/fpsyt.2019.00875

**Published:** 2019-11-28

**Authors:** Laura McKendy, Rosemary Ricciardelli

**Affiliations:** Department of Sociology, Memorial University of Newfoundland, St. John’s, NL, Canada

**Keywords:** women prisoners, desistance and probationers, mental health, recidivism, assessment

## Abstract

As a key indicator of correctional performance, returns to custody are a topic of much empirical inquiry, yet there remains considerable debate regarding questions around *who* returns and *why*, as well as the factors that support or impede successful post-release outcomes. Research examining the post-release trajectories of federal releasees in the Canadian context, particularly in the case of women, is necessary to identify opportunities for more responsive case management practices. Drawing on the case files of 43 formerly-federally-incarcerated women referred to a day reporting centre in a large Canadian city, we explore the profiles of women who returned to federal custody from those who did not, considering factors related to demographics, personal history, specifically mental health and mental health needs, static risk and dynamic need. In general, we found that those who returned to custody tended to have more needs and more complex needs relative to non-returners. Notable differences were evident in relation to criminal history, reintegration potential, dynamic factor needs, the presence of a mental health condition, the presence of substance addiction and institutional adjustment (as measured by institutional charges and segregation placements). While not attempting to present causal relationships, we shed light on the case management needs of this particular group and identify areas in need of further inquiry.

## Introduction

In assessing the performance of correctional services, a key indicator for prison administrators, management, and researchers is parolee rates of recidivism, typically referring to a return to crime or prison (1). The literature on desistance from crime and the post-release trajectory of prisoners has blossomed in many countries (2–8). However, desistance remains marked by ongoing debates, primarily around definitional matters (e.g., the meaning of “desistance”) but also theoretical questions related to how and why desistance occurs (9).

We contribute to this body of literature by analyzing the profiles of prisoners who return (returners) to prison and those who do not (non-returners) within a sample of self-identifying female former federally incarcerated prisoners under community supervision. Using case file records from the Offender Management System, we compare those who returned to custody from non-returners on a variety of risk/need measures and variables. As an exploratory analysis, we shed light on some of the factors that may be shaping post-release outcomes and the case management needs of releasees.

### Prison Release Outcomes and Returns to Custody

The literature on reintegration and re-entry has grown internationally, with scholars presenting diverse findings regarding factors that both promote and impede successful reintegration. Factors at the personal, interpersonal and structural levels have been deemed valuable in post-release outcomes (10). One set of factors that shape re-entry is tied to the crime cycle itself; i.e., criminogenic needs, otherwise known as dynamic risk factors (11). Such factors, found to be correlated with offending more generally, include substance use issues, mental health issues, negative associates, and poor attitudes/coping skills (12–17). Researchers suggest that dynamic risk may be tied to reoffending risk; for example, examining federal female releasees, Greiner, Law and Brown (18) found that scores on most domains of dynamic risk decreased among women who did not reoffend, with the domains of employment and associates having the strongest association with recidivism.

Another set of factors that can influence re-entry relates to the social and subjective effects of incarceration and criminal-justice involvement. For example, former prisoners may experience adjustment difficulties associated with the transition from institutional to free-world living (12, 19) and the stigma of the “ex-offender” label (20), which can compound difficulties finding employment (21, 22) and suitable housing (12, 21, 23). Experiences during incarceration (e.g., involvement in institutional incidents) may also be predictive when analyzing post-release outcomes, although mixed findings have been produced (24–26). In general, the impact of incarceration on subsequent offending is disputed, with researchers pointing to both criminogenic and reformative effects (27).

Paralleling findings regarding risk factors and obstacles to re-entry are findings tied to factors that support reintegration and desistance. While the subject of definitional debate, desistance is largely understood as the *process* by which those involved in crime move towards a pro-social existence (9). On this topic, scholars have emphasized the interplay between socio-structural and personal factors (28, 29). More specifically, both social factors [e.g., economic and housing opportunities; (29)] and individual level factors [e.g., a change in identity; (16, 30–33)]; are deemed pivotal to the desistance process. In regards to specific life events and processes, researchers suggest that employment, marriage, and aging are key (9).

A sub-set of literature on reintegration has focused on the specific experiences of female releasees (34–40). Women face many of the same challenges as men upon release, at both the social and individual levels (41), and may share similar predictors of recidivism (42). However, both supportive and risk factors may be gendered; for example, parenthood and family bonds may be more relevant in women’s desistance relative to that of men (41). Likewise, certain challenges may be embedded in broader gender structures (39). For example, as noted by Opsal and Foley (39), women at release may experience greater concerns related to physical and mental health, attributable, at least in part, to the higher prevalence of chronic health conditions, mental illness, and substance misuse among women. In regards to obtaining employment—a factor supportive of the desistance process (43)—women’s experiences are shaped by the consequences of a gendered labour market, such as pay inequity, discrimination, and inaccessible childcare options (39). Histories of trauma, which are common among incarcerated women (44), may also serve as an impediment to reintegration. For example, Doherty et al. (45) found that in an effort to deal with past experiences of abuse, women may return to substance use as a coping mechanism.

For both men and women, researchers have examined the effects of correctional interventions on the reintegration process. Some researchers have considered how correctional programs impact release outcomes; however, varied impacts (positive, null, negative and differential) have been reported [e.g., (46–52)]. Illustratively, (53) found that the influence of federal correctional programming is mediated by prisoner risk classification; more specifically, programs had a positive impact on moderate to high risk prisoners (evidenced by fewer segregation placements, revocations, and returns to custody), but had a negative impact on low-risk prisoners (evidenced by a greater number of institutional incidents, segregation placements, and revocations).

Another area of inquiry in the study of recidivism and desistance relates to how community supervision models and dynamics influence post-release outcomes. The impact of supervision, particularly of parole officers, on post-release outcomes is a topic of debate, with scholars noting supportive and helpful aspects on the one hand (35) and overly restrictive and punitive (and ultimately counter-productive) effects on the other [e.g. (54)]. Here, researchers have largely focused on women’s experiences; finding an emergent tension between the requirements of supervision on the one hand, and reintegration efforts on the other [e.g., 36, 55].

In summary, debate regarding the factors associated with both desistance and recidivism marks the study of reintegration. Although certain factors are largely agreed upon as supportive of desistance [e.g., aging, marriage and employment; (9)], there remains inconsistent findings regarding the influence of factors such as correctional interventions, supervision styles, and other variables on post-release outcomes. Literature on women’s experiences of reintegration has pointed to similarities with the experiences of men (41, 42) but also the ways in which re-entry is shaped by gendered factors [e.g. relating to employment, health, family and past experiences of trauma; 39, 45)]. In the current research, we further knowledge on women’s trajectories upon release from federal custody in Canada through an exploratory study examining the post-release outcomes of a community sample of women. In particular, we examine the risk/need profiles of women who returned to custody (returners) versus those who did not (non-returners) prior to their warrant expiration date (WED) and/or for a new federal sentence. Moreover, we provide insight into the mental health needs of formerly federally incarcerated women.

## Materials and Method

The case records of a community sample of 43 adult female releasees referred to the Crossroads Day Reporting Centre (CDRC) in Ontario were analyzed to explore post-release outcomes and returns to custody, and the risk/need profiles of those who return versus those who do not. The CDRC provides case management support to individuals under community supervision, including for those classified as high risk. In the study at hand, most women were referred to the CDRC when they faced issues in finding employment and/or housing; thus, they tended to be facing difficulties in such elements of community reintegration. These difficulties were undeniably influenced by geo-social factors (e.g., local housing and economic factors), although housing and employment have been identified in previous research as areas of concern among prisoners returning to communities more generally [e.g., (12, 21, 23, 29)]. It is important to clarify that this sample is not intended to be representative of the clients of the reporting centre, nor the federal community supervision population more generally. We aim instead to present a social profile of women who returned (and did not return) to custody, shedding light on their risk/need characteristics and case management needs.

Ethics approval was provided from the principle investigator’s (Ricciardelli) university Research Ethics Board and study approval was awarded from Correctional Services Canada, which enabled access to participant files. Participant consent was voluntary and acquired within the CDRC client intake processes (e.g., during entry interviews or initial contact meetings); often participants were also part of other qualitative studies conducted by the primary investigator. Participant names and identifying information were removed from any hard copies and each file was assigned an identification code. A Masterfile linking participant codes to participants was maintained for follow up purposes and to track participants to WED. Access to the Masterfile is restricted to those with Offender Management System access, appropriate security clearances, and signed agreements of confidentiality and non-disclosure. Coding for individuals involved the construction of close ended items, similar to a survey, which the participants’ files were used to complete. Coded cases were entered into survey software and subsequently exported into SPSS for statistical analysis.

Information was coded pertaining to a variety of factors starting with demographic factors and criminal profiles; including basic background factors (e.g. age, race, marital status, level of education), sentence information (e.g. index offence(s), sentence length), and criminal history (e.g. prior adult and youth convictions). Using a variety of case file documents, information related to mental health conditions (including self-reported or documented diagnoses), previous suicidal/self-injurious behaviours, and substance use history (including both alcohol and drugs) were coded. Mental health information was based on self-reports and official diagnoses as discussed in correctional documents (e.g., Correctional Plans; Criminal Profiles), decision files (e.g., Assessment for Decision documents) and casework record files. Results from the Computerized Assessment on Substance Abuse were also recorded; the item identifying a link between substance use and offending was used in the current analysis.

Information was also coded relating to a variety of risk and need measures and indicators. Dynamic needs were coded using results at intake on the Dynamic Factors Identification and Analysis and its revised version (DFIA-R),^1^ which relates to seven key domains, namely personal/emotional orientation, associates, education and employment, substance use, marital and family, attitudes, and community functioning (56). Static risk, based on criminal history, was coded using results at intake on the Static Factor Assessment (56). The accountability, motivation and reintegration potential measures [which are categorical items with possible answers of high, medium and low; 56)] were also recorded. Accountability level measures the extent to which the prisoner takes accountability for their crimes and is involved in their Correctional Plan so as to change problematic behaviors (56). Motivation level refers to the offender’s motivation to change (56), while reintegration potential relates to the level of correctional intervention needed and is assessed using the results from the Custody Rating Scale (CRS), the Static Factor Assessment and the Dynamic Factor Rating (56). Flags related to engagement with one’s correctional plan (a “yes or no” measure that is assessed by combining ratings on motivation, accountability and responsivity; 56) and the presence of responsivity issues [characteristics that impede a person’s ability to respond to correctional interventions, such as. language barriers, learning disabilities, personal/emotional factors, etc.; 56)] were also recorded. Finally, results on the CRS at intake, a tool used at intake assist in determining institutional security level, were recorded as minimum, medium or maximum (57).

To complement understanding of risk and need scores, we examined institutional experiences as measured by the factors of institutional charges, segregation placements, institutional incidents, as well institutional employment and program completion. In turning to the release experience, we recoded the nature and number of conditions placed on release, recognizing that a greater number of release conditions can make re-entry increasingly challenging for parolees (58). Finally, we included release outcomes (i.e. if a releasee returned to custody or do not prior to WED and/or for a new federal sentence), allowing us to conduct an analysis based on release outcome.

The focus of the analysis that follows is on the profile of women in the community sample, with the aim of understanding the profiles of women who returned to custody and those who did not. To this end, we conducted crosstabs with release outcome as the dependent variable. Given the relatively small sample size and non-random method of sampling, our goal is not to establish factors predictive of returns; but rather, to better understand the need profiles of returners and any differences or consistencies with those of their non-returner counterparts so as to shed light on the case management needs of this group, which in turn can inform case management practices and subsequent research.

## Results

### The Profile of Returners and Non-Returners

Of the 43 participants in our sample, 23 returned to custody, with 22 returning prior to their WED. Those who returned to custody to begin a new federal sentence had all returned prior to WED, due to parole suspensions on their first federal sentence, in all but one case. Here, the woman was held in custody until WED and therefore did not have the opportunity to return to custody prior to the completion of her sentence to commence her new federal sentence. Since the remaining 20 women did not return to federal custody, a comparison of the profiles of women was conducted, examining factors related to background information, sentence information, criminal histories, mental health and substance use histories, risk/need assessment results, institutional histories and release conditions. Given the small sample size, differences may appear exaggerated due to magnified effect of small differences. Furthermore, given that the sample was taken from a particular day reporting centre in a non-randomized manner, results are not generalizable (see **Table 1** for basic profile information of the sample).

**Table 1:** Profile information.

Characteristic	Release outcome		
Average age	Non-Returners	Returners	*Total*
	(n=20)	(*n*=23)	(*n*=43)
	30.65	30.96	30.81
	*n* (%)	*n* (%)	*n* (%)
Race			
White	3 (15.0%)	8 (34.8%)	11 (25.6%)
Indigenous	0 (0.0%)	4 (17.4%)	4 (9.3%)
Black	12 (60.0%)	7 (30.4%)	19 (44.2%)
Other	5 (25.0%)	4 (17.4%)	9 (20.9%)
Marital status			
Non-partnered	12 (60.0%)	15 (65.2%)	27 (62.8%)
Partnered	8 (40.0%)	8 (34.8%)	16 (37.2%)
Level of education			
Less than high school	8 (40.0%)	14 (60.9%)	22 (51.2%)
High school or equivalent	4 (20.0%)	2 (8.7%)	6 (14.0%)
More than high school	8 (40.0%)	7 (30.4%)	15 (34.9%)
Index offence			
Homicide-related	3 (15.0%)	0 (0.0%)	3 (7.0%)
Sexual	0 (0.0%)	1 (4.3%)	1 (2.3%)
Assault	1 (5.0%)	1 (4.3%)	2 (4.7%)
Robbery	1 (5.0%)	4 (17.4%)	5 (11.6%)
Other violent	0 (0.0%)	2 (8.7%)	2 (4.7%)
Property	0 (0.0%)	2 (8.7%)	2 (4.7%)
Drug	13 (65.0%)	10 (43.5%)	23 (53.5%)
Other non-violent	2 (10.0%)	3 (13.0%)	5 (11.6%)
Adult criminal history	6 (30.0%)	11 (47.8%)	17 (39.5%)
Youth criminal history	2 (10.0%)	8 (34.8%)	10 (23.3%)
Any mental health disorder*	8 (40.0%)	14 (60.9%)	22 (51.2%)
Mood disorder	8 (100.0%)	11 (78.6%)	19 (86.4%)
Anxiety disorder	0 (0.0%)	6 (42.9%)	6 (27.3%)
Personality disorder	0 (0.0%)	5 (35.7%)	5 (22.7%)
Other	0 (0.0%)	2 (14.3%)	2 (9.1%)
History of suicidal/self-injurious behaviour	9 (45.0%)	10 (43.5%)	19 (44.2%)
History of substance abuse	5 (25.0%)	12 (52.2%)	17 (39.5%)

*All identified mental health disorders were included; totals may therefore exceed 100%.

### Comparisons and Assessments: Risk and Need

Along many background factors, there was similarity across the two groups (e.g., age, marital status, education level). Returners were somewhat more likely to have an adult criminal history (48 versus 30%) and youth criminal history (35 versus 10%) compared to non-returners. Data from risk assessment measures was compared across the two groups. Women who returned to prison were more likely to have a CRS assessment score of “minimum” at intake (52 versus 30% respectively). They were, however, less likely to have low static risk assessment scores in comparison to their non-returner counterparts (52 versus 75%), which reflects criminal history. Returners were somewhat less likely to be ranked high in accountability compared to non-returners (39 versus 50%). On motivation to adhere to one’s correctional plan, returners were somewhat more likely to have high motivation (87 versus 75% respectively). Yet, returners were considerably less likely to be ranked as having high reintegration potential (35 verses 65%). Concerning the presence of responsivity issues (factors that impede responsivity to correctional interventions), there was minimal difference evident; 10 versus 13% of non-returners versus returners). Likewise there was little difference on the engagement measure; 80% of non-returners versus 74% of returners were engaged with their correctional plan (see **Table 2**). Such findings suggest that returners, although more likely to score high on static risk factors (i.e., criminal history) and low on accountability and on reintegration potential, tended to be motivated toward change and engaged with their correctional plan; i.e. women *do* want to change their life and are motivated toward successful desistance from crime.

**Table 2:** Risk assessments and measures

Measure	Release outcome		
	Non-Returners	Returners	*Total*
	(n=20)	(*n*=23)	(*n*=43)
	*n* (%)	*n* (%)	*n* (%)
Custody rating scale			
Maximum	0 (0.0%)	1 (4.3%)	1 (2.3%)
Medium	14 (70.0%)	10 (43.5%)	24 (55.8%)
Minimum	6 (30.0%)	12 (52.2%)	18 (41.9%)
Static factor level of need			
High	2 (10.0%)	3 (13.0%)	5 (11.6%)
Moderate	3 (15.0%)	8 (34.8%)	11 (25.6%)
Low	15 (75.0%)	12 (75.0%)	27 (62.8%)
Accountability			
High	10 (50.0%)	9 (39.1%)	19 (44.2%)
Moderate	4 (20.0%)	9 (39.1%)	13 (30.2%)
Low	3 (15.0%)	0 (0.0%)	3 (7.0%)
Not indicated	3 (15.0%)	5 (21.7%)	8 (18.6%)
Motivation			
High	15 (75.0%)	20 (87.0%)	35 (81.4%)
Moderate	4 (20.0%)	3 (13.0%)	7 (13.0%)
Not indicated	1 (13.0%)	0 (0.0%)	1 (2.3%)
Reintegration Potential			
High	13 (65.0%)	8 (34.8%)	21 (48.8%)
Moderate	5 (25.0%)	10 (43.5%)	15 (43.5%)
Low	1 (5.0%)	5 (21.7%)	6 (14.0%)
Not indicated	1 (5.0%)	0 (0.0%)	1 (2.3%)
Responsivity issues	2 (10.0%)	3 (13.0%)	5 (11.6%)
Engaged in correctional plan	16 (80.0%)	17 (73.9%)	33 (76.7%)

Comparing returner and non-returner participant mental health data, women who returned to custody were more likely to have at least one mental health condition (61 versus 40% respectively). Moreover, anxiety disorders (e.g. generalized anxiety, post-traumatic stress disorder) and personality disorders (e.g. borderline personality disorder, anti-social personality disorder, psychopathy) in particular were more common among returners, while mood disorders (e.g. depression, bipolar) were prominent among both groups. Little difference was evident when it came to history of suicidal/self-injurious behaviour; the prevalence was 44% for returners and 45% for non-returners; thus revealing that nearly half of the women (*n* = 19; 44%) have histories of self-harm. Returners, however, were more likely to have histories of substance misuse in comparison to non-returners (52 versus 25%) (see **Table 1**). Although the mental health needs of all participants require directed attention and intervention, findings here highlight that returners are more likely to have a major mental disorder and/or a history of substance misuse, in line with previous research emphasizing addiction as a barrier to reintegration (14, 39, 45).

When it came to dynamic needs, returners were more likely to be as scored as having “high” overall dynamic need compared to non-returners (35 versus 10%). To analyze differences across the seven domains, we collapsed responses into two categories: (1) high/moderate; and (2) low/no need/asset to community. Given the small sample size, we did so to ensure there were sufficient data in cells for comparisons to be drawn across the two groups. Findings reveal differences in most domains. Returners were more likely to rank high or medium compared to non-returners on six of the seven domains: education/employment (74 versus 45%, returners versus non-returners); personal/emotional (74 versus 45%); substance abuse (39 versus 10%); marital/family (52 versus 15%); associates (61 versus 45%); community functioning (39 versus 20%). In the remaining domain, attitudes, there was minimal difference across the groups. For the sample at hand, dynamic needs were a factor were the most apparent differences emerged between returners and non-returners (see **Table 3**).

**Table 3:** Dynamic need domains

Characteristic	Release outcome		
	Non-returners	Returners	*Total*
	(n=20)	(*n*=23)	(*n*=43)
	*n* (%)	*n* (%)	*n* (%)
Overall Level of Dynamic Need			
High/Considerable	2 (10.0%)	8 (34.8%)	10 (23.3%)
Moderate/Some	7 (35.0%)	10 (43.5%)	17 (39.5%)
Low/No/Asset	11 (55.0%)	5 (21.7%)	16 (37.2%)
Education/Employment			
High or Moderate	9 (45.0%)	17 (73.9%)	26 (60.5%)
Low/No/Asset	11 (55.0%)	6 (26.1%)	17 (39.5%)
Personal/Emotional			
High or Moderate	9 (45.0%)	17 (73.9%)	26 (60.5%)
Low/No/Asset	11 (55.0%)	5 (21.7%)	16 (37.2%)
Not indicated	0 (0.0%)	1 (4.3%)	1 (2.3%)
Substance abuse			
High or Moderate	2 (10.0%)	9 (39.1%)	11 (25.6%)
Low/No/Asset	18 (90.0%)	14 (60.9%)	32 (74.4%)
Marital/Family			
High or Moderate	3 (15.0%)	12 (52.2%)	15 (34.9%)
Low/No/Asset	17 (85.0%)	11 (47.8%)	28 (65.1%)
Attitudes			
High or Moderate	4 (20.0%)	4 (17.4%)	8 (18.6%)
Low/No/Asset	16 (80.0%)	18 (78.3%)	34 (79.1%)
Not indicated	0 (0.0%)	1 (4.3%)	1 (2.3%)
Associates			
High or Moderate	9 (45.0%)	14 (60.9%)	23 (53.5%)
Low/No/Asset	11 (55.0%)	8 (34.8%)	19 (44.2%)
Not indicated	0 (0.0%)	1 (4.3%)	1 (2.3%
Community Functioning			
High or Moderate	4 (20.0%)	9 (20.0%)	13 (30.2%)
Low/No/Asset	16 (80.0%)	13 (56.5%)	29 (67.4%)
Not indicated	0 (0.0%)	1 (4.3%)	1 (2.3%)

We also considered which dynamic need domains were identified as “contributing factors” to the crime cycle, as noted in the dynamic factor assessment. Returners were more likely than non-returners to have five of the seven domains identified as a contributing factor, including: education/employment (26 versus 15% for returners versus non-returners), personal/emotional (91 versus 80%), substance abuse (30 versus 10%), marital/family (17 versus 10%), and attitudes (13 versus 10%). Non-returners were slightly more likely to have associates listed as a contributing factor (85 and 78% for non-returners and returners respectively) and community functioning was equally assessed as a contributing factor for returners and non-returners (35%). Again, challenges with education and employment predict returning to prison, however it is the contributing factor of substance abuse in the crime cycle that appears to be most associated with return to custody in the study at hand.

### Institutional Histories and Conditions of Release

The institutional experiences of returners and non-returners were explored to consider if and how program completion was associated with post-release outcomes; we examined if institutional adjustment issues were associated with returns to custody, as some previous studies have indicated (26). We also compared the conditions parolees must adhere to on release as awarded to returners versus non-returners.

When it came to program completion, returners were less likely than non-returners to have completed the Women Offender Engagement Program/Aboriginal Women Offender Engagement Program (WOEP/AWOEP WOEP/AWOEP; 65 versus 85%) and the Women’s Moderate Intensity Program (WMIP; 35 versus 50%). Reflecting on overall educational programs completed in prison and holding institutional employment, returners were more likely to have held institutional employment (91 versus 75%; returners versus non-returners) although educational program completion was similar for the two groups (i.e. 44 versus 40%). The impact of each is difficult to discern given it cannot be noted if their return was delayed due to their program participation or the consequence of not having completed the programming given programming is found to positively inform desistance (59, 60).

Institutional histories, measured by segregation placements, institutional charges and incidents were also examined. Returners in the sample appeared to have more tumultuous institutional histories compared to their non-returner counter-parts; for example, they were somewhat more likely to be placed in segregation (39 versus 20%, returners versus non-returners) and to have institutional charges related to disobeying rules or orders (74 versus 40%) or possessing contraband/unauthorized items (44 versus 10%). However, non-returners and returners were equally likely to have been involved^2^ in institutional incidents tied to disciplinary issues (70% for both groups). In general, the institutional adjustment concerns noted among returners falls in line with previous research pointing to an association between institutional adjustment and post-release outcomes (26).

The conditions attached to release from federal prison were analyzed for 42 of the 43 women.^3^ When released into the community on supervision (parole or statutory release), women had an average of 4.71 conditions (median = 5). Common conditions were related to substance abstinence, general or specific no-contact orders, and mental health treatment or counselling. In terms of differences in conditions across the two groups, returners were notably more likely to have a condition related to mental health treatment/counselling (55 versus 35%). Given the greater likelihood of addiction and mental health concerns among returned women, such findings are not surprising.

## Discussion and Conclusion

Our study analyzed the release outcomes of federally incarcerated women recruited in the community using participants’ case file records. Overall, about half of women returned to custody. In general, we found that those who returned to custody tended to have greater and complex needs relative to non-returners. Differences were evident in relation to criminal history, reintegration potential, dynamic factor needs, the presence of a mental health condition, the presence of substance addiction and institutional adjustment (as measured by institutional charges and segregation placements). Our findings are consistent with previous research noting a connection between criminal history and recidivism (61). In addition to criminal history and static risk, we found differences related to dynamic factors and overall needs, which fits in line with previous researching noting the link between dynamic needs and recidivism (18).

Mental health factors were also examined; around half of the sample had some type of mental health condition (61% of returners and 40% non-returners), pointing to the importance of mental health considerations in case management. Substance use issues were also more common among returners; this is not surprising given previous researchers highlight how addiction serves as a barrier to reintegration (39, 45). As evidenced by supervision conditions related to mental health treatment and substance use, the realm of supervision extends into the domain of mental health, with implications for the social service role of parole officers and case management staff. Balancing the dual function of supervision and social service delivery (62), particularly as it pertains to mental health, is an area in need of further inquiry.

The finding that women in both groups tended to have high motivation and be engaged with their correctional plan suggests that women who return to custody may have the intention and motivation for desistance; i.e., the subjective component deemed integral to personal change (9). This lends weight to the argument that desistance requires not only reformed subjective orientations, but conducive social conditions—or a “hook for change” (63).

We also noted differences between returners and non-returners institutional experiences and histories. Returners appeared to have greater institutional adjustment issues as measured by charges and segregation placements. A plausible explanation is that the factors driving the crime cycle may similarly affect institutional adjustment. Further research that analyzes the association between institutional and community adjustment is warranted, particularly given the link between institutional adjustment and recidivism remains marked by competing findings (24–26, 64). We advocate for researchers to examine the unintended effects of carceral living on women’s reintegration.

Methodological limitations impede our study from offering predictive insights on the factors that differentiate returners from non-returners. Our small sample size and non-randomized, regionally-specific sample prevent us from offering statements regarding the correlates of successful and unsuccessful post-release outcomes. Nonetheless, our research sheds light on some of the factors that may differentiate the profiles of returners from non-returners among a sample of women, and discuss the case management needs of this group. The insights derived from our study may direct subsequent empirical attention; in particular, we propose future researchers examine more closely the connection between mental health and revocations and returns, as well as the ways in which institutional experiences (including both positive and negative components) influence the post-release experience. Relatedly, we identify a need for research that seeks to better understand how formal and informal interventions in both the custodial and community settings may affect post-release success.

## Data Availability Statement

The datasets generated for this study will not be made publicly available as it contains confidential information.

## Ethics Statement

This study was reviewed and approved by the York University Research Ethics Board and the Memorial University of Newfoundland Research Ethics Board. Written informed consent was obtained from all participants.

## Author Contributions

The authors equally contributed to the article.

## Conflict of Interest

The authors declare that the research was conducted in the absence of any commercial or financial relationships that could be construed as a potential conflict of interest.

## Footnotes

1 Women in this study varied in terms of which dynamic need assessment tool was employed depending on their admission date. Overall ratings in domains were hence based on the results of either the DFIA or DFIA-R.

2 Involvement qualifiers include: victim, instigator, associate, intervener/assist victim and unknown.

3 One woman who was released at WED and subsequently returned for a new federal sentence is not included in this analysis.
